# Spatial Scale Effects of the Relationship between Fractional Vegetation Coverage and Land Surface Temperature in Horqin Sandy Land, North China

**DOI:** 10.3390/s21206914

**Published:** 2021-10-19

**Authors:** Rongrong Qiao, Chunyuan Dong, Shuxin Ji, Xueli Chang

**Affiliations:** School of resources and environmental engineering, Ludong University, Yantai 264025, China; rongrongqiao@m.ldu.edu.cn (R.Q.); chunyuandong@m.ldu.edu.cn (C.D.); xinshuji@m.ldu.edu.cn (S.J.)

**Keywords:** fractional vegetation cover, land surface temperature, landscape pattern metrics, thermal infrared, meteorology, correlation analysis, Horqin

## Abstract

Sandy range land refers to a major component of grassland area types in the semi-arid area of northern China. Monitoring of vegetation and land surface temperature (LST) using remote sensing technology can help determine the degree of desertification in a regional and/or sub regional scale, as in the Horqin Sandy Land selected in this study. Correlation analysis was performed to examine the relationship between the fractional vegetation coverage (FVC) and the LST within one growing season (from May to August 2017), at different spatial scales. The results showed that the FVC increased from 0.12 in May to 0.29 in August, and the LST increased first and then declined. The highest LST was 41.68 °C in July, while the lowest was 28.62 °C in August. At the grid scale, the LST increased first and then declined with the increase of the FVC on 25 May, 10 June, and 29 August; the FVC ranged from 0.29–0.38, 0.27–0.32, and 0.29–0.38 with the preference of the ‘turning point’, respectively. A negative correlation was identified between the FVC and the LST and without any ‘turning point’ in the fitting curve on 28 July. The correlation between FVC and LST complied with the grid scale at the sample area scale. The coupling analysis of landscape pattern expressed by FVC and LST showed that, the landscape evenness, Euclidean nearest neighbor distance, and landscape splitting degree all showed strong coupling correlation in any study period (P). The landscape aggregation of FVC and LST showed a good coupling at the relatively high and low air temperature conditions of P1 and P3. Landscape contagion showed a good coupling between FVC and LST at relatively moderate air temperature condition of P1 and P4. Air temperature conditions and characteristics of vegetation coverage should be considered for a more targeted analysis when analyzing the relationship between FVC and LST and attention should be paid to the timing and type of study area in practical application.

## 1. Introduction

The degradation of ecosystems caused by land desertification threatens the ecosystem health of the world [1]. The arid and semi-arid areas of northern China are prone to desertification and related eco-risks, such as sandstorms, while spring is the high incidence period [2,3]. Land surface temperature (LST) and vegetation are important surface indexes in sandstorm monitoring [4,5], and their relationship has important indicative significance in the present status of desertification with different scales and hierarchical structures [6,7]. LST is an important parameter indicating the exchange of matter and energy between land and atmosphere, and the change of ecological environment [8,9]. It contributes to the study on eco-hydrology, drought monitoring, and global changes [10,11]. With the development of remote sensing technology, large-scale ground temperature can be easily obtained by thermal infrared band. The methods of obtaining LST by spaceborne thermal infrared sensor involves single-channel algorithm, multi-channel algorithm, multi-angle algorithm, and so on [12,13,14,15]. Landsat series data have obvious advantages in time continuity and data quality in the existing thermal infrared data, so they have been widely used to extract LST, especially with Landsat 8 [16,17].

The LST is affected by numerous factors such as the composition of the surface material and the properties of the underlying surface. As an important participant in the surface ecological process, vegetation affects the acceptance of solar radiation, so it has a complex impact on the distribution pattern of LST [18,19]. The effect of vegetation on LST could be got conveniently and effectively by constructing vegetation characteristic parameters (e.g., Normalized Difference Vegetation Index (NDVI), Fractional Vegetation Coverage (FVC)). Weng et al. [20] investigated the correlation between NDVI, FVC, and LST on seven types of land coverage (e.g., commercial, residential and cropland) at a pixel scale. The results showed that FVC was more correlated than NDVI with LST. Amiri et al. [21] investigated the temporal variability of vegetation coverage and LST by building a temperature vegetation index in a rapidly developing city, Tabriz metropolitan area in Iran. Chakraborty and Lee [22] estimated the difference of the surface urban heat island (UHI) intensity and the seasonal control of vegetation on UHI at a global scale by adopting a new simplified algorithm. There are also studies on the relationship between vegetation and climate response based on remote sensing long-time series data [23,24]. Notably, the mentioned studies concentrated on the temporal and spatial distribution characteristics of vegetation, LST and UHI, as well as the impact of urban landscape on the surface thermal environment [25,26,27,28]. Accordingly, what is the corresponding relationship between FVC and LST in a single surface cover area, and whether the distribution pattern of LST will show diversity characteristics? Sandy range land have been widely distributed in the arid and semi-arid areas of Eurasia. They are vital places for animal husbandry activities, as well as relatively serious desertification-prone areas in Eurasia. In the study of desertification, vegetation coverage can reflect the variations of surface ecological environment directly and underpin the division of different stages of desertification [25,26,27,28,29,30]. The vegetation in semi-arid sandy area is sparse and mostly distributed in patches, which is obviously different from the urban area [31,32]. Therefore, it needs to be proved whether the conclusion in urban area can be directly used to explain the correlation between FVC and LST in semi-arid sandy land [20,33].

Horqin Sandy Land is located at the passing area of sandstorms affecting northeast China, and it refers to a typical ecologically fragile area in the agro-pastoral ecotone of northern China as well. Over the past 40 years, development and reversal of desertification have changed frequently in this region. The scientific problems to be solved include (1) correlation between FVC and LST at different spatial scales and meteorological conditions, and (2) spatial coupling characteristics of landscape pattern between FVC and LST.

## 2. Materials and Methods

### 2.1. Study Area

**Figure 1:**
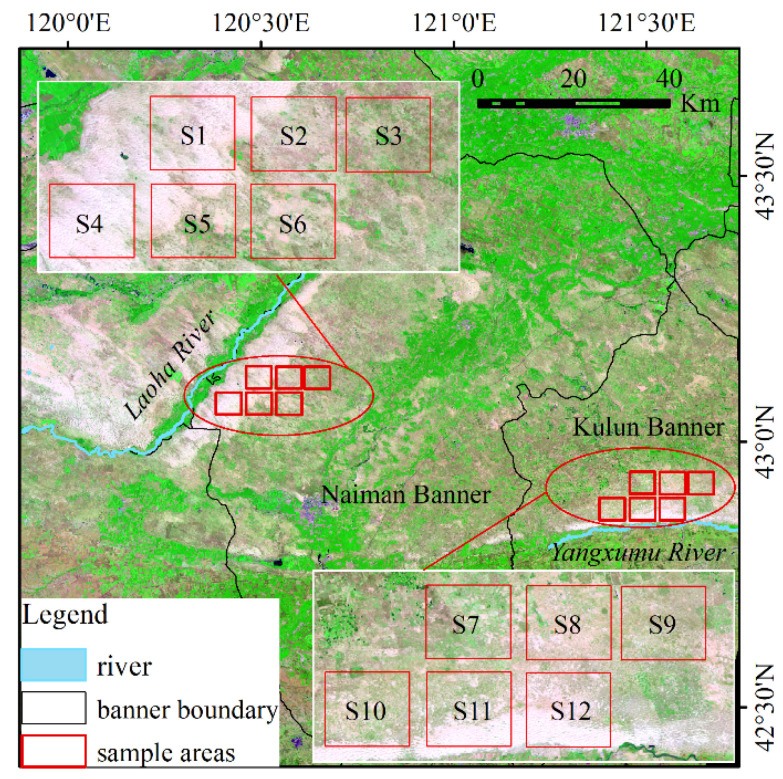
Location of the study area.

Horqin Sandy Land is located in the southeast of Inner Mongolia Autonomous region, northern China, with a latitude of 43°2′ N and longitude of 120°25′ E, one of the areas most seriously affected by desertification in China. This area pertains to temperate semi-arid continental monsoon climate. To be specific, the eolian sandy soil is widely distributed in this area, the average annual temperature is nearly 6 °C, and the average annual precipitation is about 350 mm, mainly in summer. The vegetation patterns are patchy primarily, composed of *Agriophyllum squarrosum*, *Corispermum hyssopifolium* L., *Coragana Microphylla*, *Artemisia halodendrom*, *A. frigida*, *Cleistogenes squarrosa*, etc. The sample areas of this study include Naiman Banner and Kulun Banner in the central and southern part of Horqin Sandy Land, located in the east bank of Laoha River and the north bank of Yangxumu River, respectively, which are typical areas of sandy range land in Horqin (Figure 1). On the background of sandy land (e.g., mobile dunes, semi-mobile dunes, fixed dunes, and semi-fixed dunes), 12 rectangular sample areas with an equal area (24 square kilometers) were selected (Figure 1 and S1–S12 in Figure S1).

### 2.2. Data

Landsat 8 satellite exhibits the flexibility of regional monitoring, which carries OLI (Operational Land Imager) and TIRS (Thermal Infrared Sensor) sensors. The spatial resolution of Band1–Band9 is 30 m (Band8 is 15 m), and that of Band10–Band11 is 100 m. Since this study should explain the correlation between FVC and LST at different temperature conditions, cloud free screening was performed on the USGS website (https://earthexplorer.usgs.gov/, accessed on 31 December 2020), and a continuous growing season time series was taken to make the data comparable. Finally, four images (path: 121, Line: 30) ingested on 25 May 2017, 10 June 2017, 28 July 2017, and 29 August 2017 were selected. After the image acquisition, the information could be extracted through the pre-processing (e.g., radiation correction and atmospheric correction), in which OLI images were employed for vegetation information extraction. Furthermore, TIRS images were adopted to extract LST data.

Table 1 lists the meteorological data observed on the same day of images acquisition by the National Field Scientific Observation and Research Station of Naiman farmland ecosystem and the National Meteorological Observatory of Kulun Banner in Inner Mongolia Autonomous Region. In Table 1, “air temperature” was measured at 10:40 am consisting with the acquisition time of Landsat 8 scene. “Maximum sLST” and “minimum sLST” were taken from the maximum and minimum values of four ground temperature measurements at 2:00, 8:00, 14:00, and 20:00 in a day, respectively.

### 2.3. Methodology

#### 2.3.1. Extraction for FVC

Pixel dichotomy were used to extract fractional vegetation coverage:

(1) $$\mathrm{FVC}=\left[\left(\mathrm{NDVI}\;-{\;\mathrm{NDVI}}_{\mathrm{Soil}}\right)/\left({\mathrm{NDVI}}_{\mathrm{Veg}}-{\;\mathrm{NDVI}}_{\mathrm{Soil}}\right)\right]$$

(2) $$\mathrm{NDVI}=\frac{\rho_{nir}-\rho_{r}}{\rho_{nir}+\rho_{r}}$$

where, ρ*_nir_* and ρ*_r_* denote reflectance of near infrared and red bands, respectively. NDVI_soil_ is the NDVI of bare sand or no vegetation coverage pixel, and NDVI_veg_ represents the NDVI of the pixel completely covered by vegetation, i.e., the NDVI of pure vegetation pixel. In this study, the field measured value in Horqin Sandy Land was taken (measured by ASD FieldSpec 4), i.e., NDVI_veg_ = 0.75, NDVI_soil_ = 0.12.

#### 2.3.2. Extraction for LST

The Landsat 8 TIRS sensor comprises two thermal infrared channels (B10 and B11), whereas the USGS official website highlights that the TIRS B11 band exhibits some instability, so the split window algorithm is not recommended. For this reason, this study selected the Radiative Transfer Equation (the atmospheric correction method) to retrieve LST based on B10 band. The radiative transfer equation complies with a basic theory that the radiance value received by the satellite sensor largely consists of (1) atmospheric upstream radiation, (2) the energy reflected by the atmospheric downward radiation after reaching the ground, as well as (3) the energy of the real radiation from the ground to the satellite sensor after passing through the atmosphere.

(3) $$L_{\lambda }=\left[\varepsilon B\left(T_{S}\right)+\left(1-\varepsilon \right)L↓\right]\tau +L↑$$

where, L_λ_ denotes thermal infrared radiance received by satellite sensors, B(T_s_) expresses blackbody radiation, the atmospheric correction parameters L↓, τ, L↑ represent the upwelling radiance, atmospheric transmittance, and the downwelling radiance, respectively. They can be originated from the website (http://atmcorr.gsfc.nasa.gov/, accessed on 31 December 2020) presented by NASA through entering the acquisition time of imagery and the center latitude, longitude. ε represents the surface emissivity calculated by employing the methods of sobrino et al. [34]. T_s_ can be finally obtained by inverting Planck’s laws as:

(4) $$T_{s}=\frac{K_{2}}{\ln\left(\frac{K_{1}}{\left[L_{\lambda }-\;L↑-\tau \left(1-\varepsilon \right)L↓\right]/\tau \varepsilon }+1\right)}$$

(5) $${\mathrm{LST}=T}_{S}-273$$

where, K_1_ = 774.89 W/(m2·µm·sr), K_2_ = 1321.08 K for Landsat 8 TIRS band10. T_S_ and LST denote the land surface temperature in K, °C, respectively.

#### 2.3.3. Grading of FVC and LST

**Figure 2:**
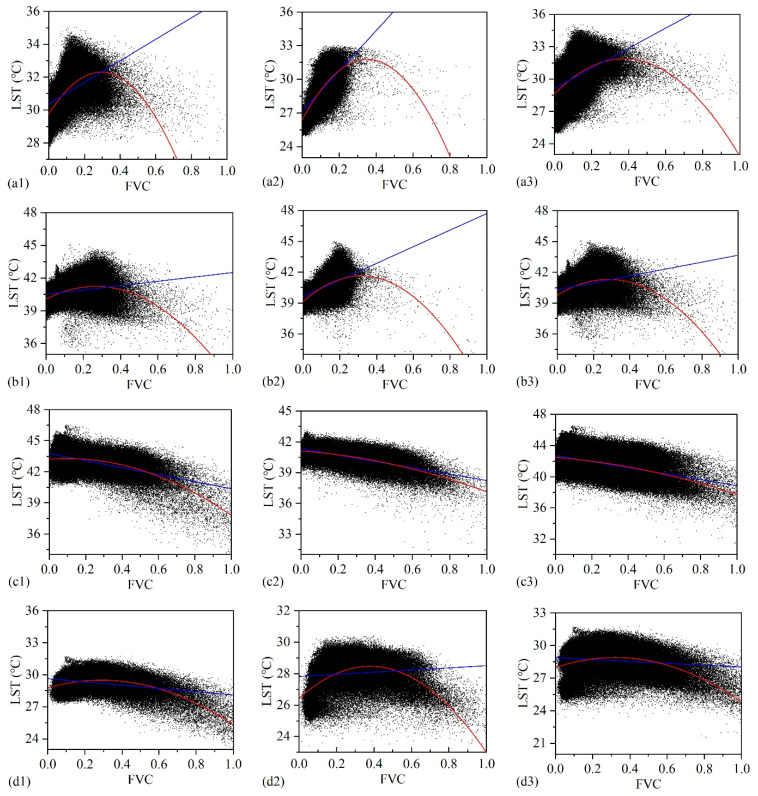
Relationship between FVC and LST at grid scale on P1–P4 ((**a**), P1; (**b**), P2; (**c**), P3; (**d**), P4. (**1**), S1–S6; (**2**), S7–S12; (**3**), S1–S12. The blue line is linear fitting line, and the red line is binomial fitting line between FVC and LST.).

As impacted by the limitation of the quality of remote sensing data, image of 25 May, 10 June, 28 July, 29 August 2017 of Landsat 8 were selected for the analysis. This group of data were obtained from the different stages of growing, as well as at different temperature conditions. The acquisition time of the four periods of the remote sensing data was named by P1, P2, P3, and P4 (Table 1), respectively. Moreover, the correlation between FVC and LST was analyzed from the grid and sample area scales, where the grid scale is analyzed from three groups: (1) the sandy land on the east bank of the Laoha River (S1–S6 in Figure 1), (2) the sandy land on the north bank of the Yangxumu River (S7–S12 in Figure 1), as well as (3) overall (S1–S12 in Figure 1).

To investigate the landscape pattern correlation between FVC and LST, they should be graded. Given the scarcity of vegetation in sandy areas and the independence of four meteorological conditions, FVC and LST fall to four grades (Table 2) based on the method of mean standard deviation [35]. In addition, since the spatial resolution of OLI images was 30 m, and the resolution of TIRS images was 100 m, both FVC and LST images were resampled to 100 m before the classification according to the minimum bucket rule [36].

#### 2.3.4. Selection and Analysis of Landscape Metrics

Landscape metrics is capable of indicating the spatial structure and distribution characteristics of landscape patterns. Based on Fragstats software, the corresponding landscape metrics were selected for quantity, shape, proximity, uniformity, and aggregation, and the correlation test with landscape metrics of FVC and LST was performed. The extremely high autocorrelation metrics (*r* ≥ 0.87) were removed. Eventually, landscape pattern metrics selected seen in Table 3.

According to the landscape pattern analysis of FVC and LST, the sample areas with the smallest, largest, and closest to the mean FVC were selected from P1–P4 as the representative sample areas for the analysis (Table 4).

Correlation coefficient (*r*) expresses the linear correlation between FVC and LST and their landscape pattern metrics. If *r* > 0, there would be a positive correlation between FVC and LST and their landscape pattern metrics; *r* < 0, there would be a negative correlation. The closer the absolute value of *r* to 1, the stronger the correlation would be, the closer the absolute value of *r* to 0, the weaker the correlation would be.

(6) $$r_{xy}=\frac{\sum_{i=1}^{n}\left(x_{i}-\bar{x}\right)\left(y_{i}-\bar{y}\right)}{\sqrt{\sum_{i=1}^{n}{\left(x_{i}-\bar{x}\right)}^{2}}\sqrt{\sum_{i=1}^{n}{\left(y_{i}-\bar{y}\right)}^{2}}}$$

where, *x*, *y* denote the value of FVC and LST at the grid scale and the sample area scale, or the landscape pattern metrics of FVC and LST on the landscape scale. To be specific, *n* denotes the number of samples, and $\bar{x}$, $\bar{y}$ express the average value of *x*, *y* samples, respectively.

Coefficient of determination (R^2^) is also known as goodness of fit. As revealed from the fitting analysis of FVC and LST polynomials, the larger the R^2^, the higher the degree of FVC’s interpretation of LST will be.

The coefficient of variation (*CV*) is capable of eliminating the dimensional influence and comparing the degree of discrete variation of the data.

(7) $$CV=\frac{S}{\bar{x}}\times 100\%$$

where, *S* denotes the standard deviation of a set of FVC or LST, and $\bar{x}$ represents the average of that set of data. In this study, the larger the *CV*, the greater the degree of discrete variation of FVC and LST will be between or within groups, and the more obvious the gradient will be. *CV* ranges from 0–15% for small variation, 16–35% for moderate variation, >36% for high variation.

## 3. Results

### 3.1. Basic Characteristics of FVC and LST in Sample Areas

As indicated from the basic characteristics of FVC in the study area (Table 5), the average FVC of the sample areas (S1–S12) was 0.12, 0.15, 0.27, and 0.29 at P1–P4, respectively, and the overall vegetation coverage of the study area was at a low level. The *CV* of average FVC of the whole study area (S1–S12) from P1 to P4 was 40.1%. At P1, the difference between the maximum sample area and the minimum sample area of FVC was 0.16 and the *CV* of FVC between S1–S12 was 34.3%. At P2, the difference between the maximum sample area and the minimum sample area of FVC was 0.21, and the *CV* of FVC between 12 sample areas was 37.0%. At P3, the difference between the maximum sample area and the minimum sample area of FVC was 0.32, and the *CV* of the 12 sample areas was 37.7%. At P4, the maximum sample area and the minimum sample area of FVC had a difference of 0.33, and the *CV* of the 12 sample areas was 33.5%. At P1 and P4, FVC showed moderate variability of the 12 sample areas, but showed high variability at P3 and P4. Overall, FVC achieved a certain degree of gradient between S1–S12 at P1–P4.

During the study period, the average LST of the sample areas at P1-P4 was 30.18, 40.80, 41.68, and 28.62 °C, respectively (Table 5), and the *CV* of the average LST of the whole study area from P1 to P4 was 19.47%. Specifically, the variation coefficient of LST of the 12 sample areas was the largest at P1, which was 4.2%; the *CV* of LST was the smallest at P2, which was 1.6%. The overall average LST was peaked at P3 in the study area, and the *CV* of LST of the S1–S12 was 3.2%; at P4, the overall average LST in the study area was the minimum, and the *CV* of LST between S1–S12 was 2.7%. On a whole, the LST of P1–P4 was at a medium level of variation, and the LST of the 12 sample areas at P1–P4 varied overall at small levels.

### 3.2. Relationship between Vegetation Coverage and Land Surface Temperature at Grid Scale

According to the linear and binomial fitting results of FVC and LST, the FVC of S1–S6 showed a positive correlation (*r* = 0.49) with LST at P1. According to the results of binomial fitting (R^2^ = 0.43), the fitting curve showed a turning point when FVC = 0.29. That was, when FVC > 0.29, LST decreased with the increase in FVC (Figure 2(a1)). FVC of S7–S12 showed a positive correlation (*r* = 0.64) with LST. As revealed from the results of binomial fitting (R^2^ = 0.50), when FVC = 0.35, the fitting curve showed a turning point, and when FVC > 0.35, LST decreased with the increase in FVC (Figure 2(a2)). The overall results of S1–S12 were consistent with the results of the first two groups, a positive correlation was identified between LST and FVC (*r* = 0.43). The binomial fitting (R^2^ = 0.23) showed that when FVC = 0.38, there was a turning point, LST began to decrease with the increasing FVC when FVC > 0.38 (Figure 2(a3)).

At P2, LST was positively correlated with FVC on S1–S6 (*r* = 0.22). As indicated from the results of binomial fitting (R^2^ = 0.18), there was a turning point when FVC = 0.27, and LST began to decrease with the increase of FVC when FVC > 0.27 (Figure 2(b1)). Also, a positive correlation was found (*r* = 0.47) between LST and FVC on S7–S12. According to the results of binomial fitting (R^2^ = 0.28), the fitting curve showed a turning point when FVC = 0.32, and LST began to decrease with the increase of FVC when FVC > 0.32 (Figure 2(b2)). The results obtained by S1–S12 complied with the first two sets of data, a positive correlation was reported between LST and FVC (*r* = 0.30). As suggested from the results of binomial fitting (R^2^ = 0.20), the fitting line had a turning point when FVC = 0.28, LST began to decrease with the increase in FVC when FVC > 0.28 (Figure 2(b3)).

At P3, a negative correlation was identified (*r* = 0.54) between LST and FVC on S1–S6, and the binomial fitting result did not show any turning point (Figure 2(c1)); a negative correlation was identified (*r* = 0.61) between LST and FVC on S7–S12, and the binomial fitting result showed that no turning point (Figure 2(c2)). The results obtained by S1–S12 complied with those before, and a negative correlation was identified between LST and FVC (*r* = 0.42), and the binomial fitting results showed that LST decreased with the increase of FVC without any turning point (Figure 2(c3)).

At P4, a negative correlation was reported between LST and FVC in S1–S6 (*r* = 0.32). According to the result of binomial fitting (R^2^ = 0.32), there was a turning point when FVC = 0.29, LST decreased with the increase of FVC when FVC > 0.29 (Figure 2(d1)). A positive correlation was reported between FVC and LST in S7-S12 (*r* = 0.11). In addition, as indicated from the results of binomial fitting (R^2^ = 0.29), the fitting curve showed a turning point when FVC = 0.38, and LST tended to decrease with the increase of FVC when FVC > 0.38 (Figure 2(d2)). On the whole, there was a negative correlation (*r* = 0.13) between LST and FVC in S1–S12. As revealed from the results of binomial fitting (R^2^ = 0.14), the fitting curve showed a turning point when FVC = 0.32, and LST decreased with the increase in FVC when FVC > 0.32 (Figure 2(d3)).

### 3.3. Relationship between FVC and LST at Sample Area Scale

**Figure 3:**
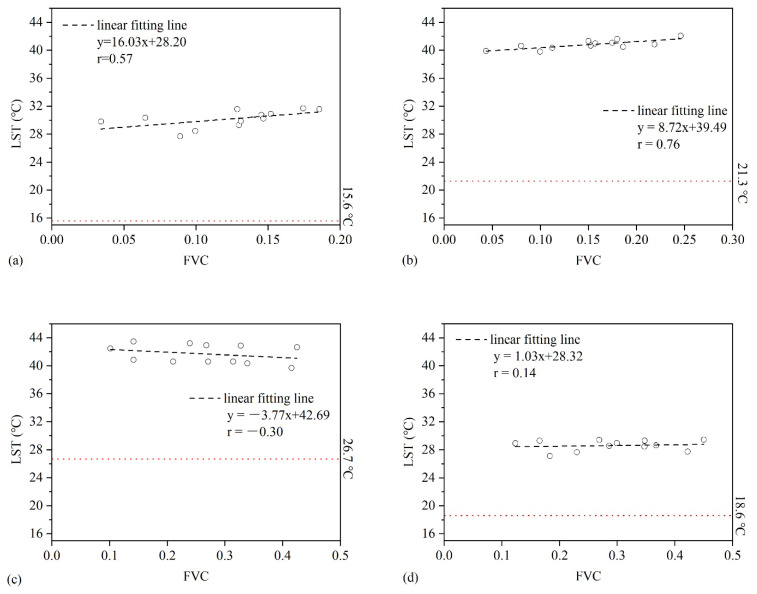
Relationship between FVC and LST at sample area scale on P1–P4 ((**a**), P1; (**b**), P2; (**c**), P3; (**d**), P4. The red dotted line is the average air temperature observed by Naiman and Kulun field stations on the same day).

As suggested from the correlation analysis between FVC and LST at the sample area scale (Figure 3), the effect of FVC on LST at P1–P4 was different. At P1, P2, and P4, FVC and LST showed a positive correlation. To be specific, the average air temperature in P2 was the highest among P1, P2, and P4, which was 21.3 °C, and the correlation coefficient (r = 0.76) between FVC and LST reached the highest. At P3, a negative correlation was found between FVC and LST (r = −0.30), and the average air temperature in P3 was the highest among P1–P4.

### 3.4. Analysis of FVC and LST Pattern on Landscape Scale

On the landscape metrics selected by Table 3, the landscape pattern metrics correlation analysis of FVC and LST at P1–P4 revealed a significant correlation (Table 6) in any representative sample area (Table 4). Among the 12 representative sample areas in Table 6, the least correlation coefficient was reported in P1 S3, which was 0.690. The maximum correlation coefficient was identified in the P4 S4, which was 0.996.

**Figure 4:**
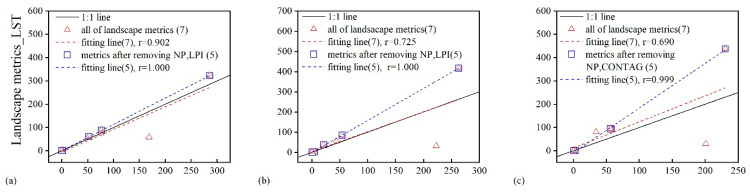

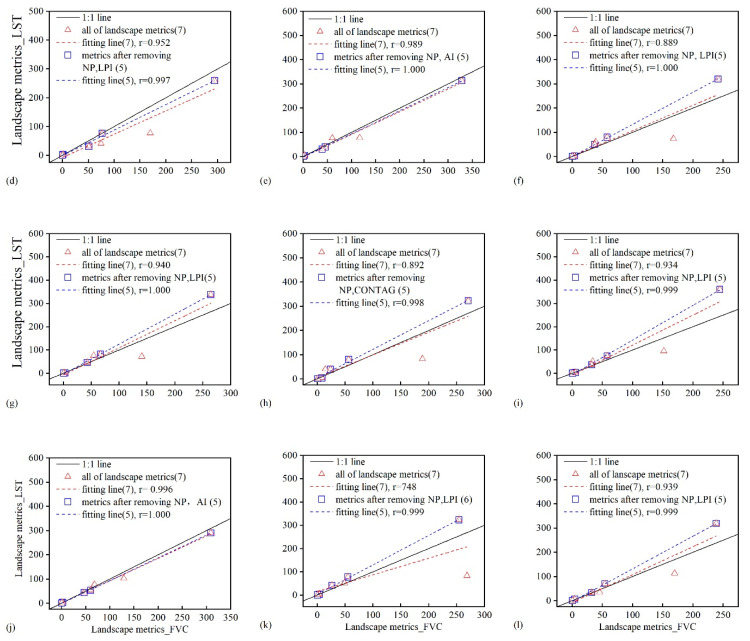
Relationship of landscape pattern metrics between FVC and LST at representative sample area ((**a**), P1, S4; (**b**), P1, S2; (**c**), P1, S3; (**d**), P2, S4; (**e**), P2, S10; (**f**), P2, S3; (**g**), P3, S4; (**h**), P3, S5; (**i**), P3, S3; (**j**), P4, S4; (**k**), P4, S8; (**l**), P4, S3).

In the sample area with maximum correlation coefficient (Figure 4j), whether all seven metrics in Table 3 were used or the discrete larger metrics (NP and AI) were excluded, FVC and LST landscape pattern metrics were closely correlated (the red fitting dotted line, seven metrics; the blue fitting dotted line, five metrics), the correlation coefficients reached 0.996 and 1.000, respectively. In the sample area with minimum correlation coefficient (Figure 4c), the landscape pattern metrics of FVC and LST showed a close relation by employing the seven metrics in Table 3 with a correlation coefficient of 0.690 (the red fitting dotted line, seven metrics), and the correlation coefficient was 0.999 after excluding the larger discrete metrics NP and CONTAG (the blue fitting dotted line, five metrics).

As indicated from the comparison of FVC and LST landscape metrics fitting analysis of all sample areas in Table 6, Figure 4, SHEI and ENN_MN, SPLIT index showed a good fit in any representative sample area at P1–P4, while NP index led to a smaller correlation coefficient between FVC and LST landscape metrics in all representative sample areas.

To be specific, there were some phenomena that the landscape metrics was only suitable for specific conditions. At P1, AI index had a good correlation in the fitting of FVC and LST landscape pattern metrics, while LPI and CONTAG index caused the smaller fitting correlation coefficient (Figure 4a). At P2, CONTAG index also showed good fit, while LPI and AI index decreased correlation coefficient of the fitting (Figure 4d–f). At P3, the AI index showed a good fitting, and the fitting correlation coefficient was smaller as impacted by the LPI and CONTAG index (Figure 4g–i). At P4, CONTAG index showed a good fitting, while LPI and AI index caused the fitting correlation coefficient to reduce (Figure 4j–l).

## 4. Discussion

The effect of FVC on LST refers to a common phenomenon, and there have been considerable research reports in different regions and different types of surface cover [37,38]. Moreover, the reports emphasized that the temporal and spatial distribution characteristics of LST was the result of many factors, and the seasonal difference of LST was also proved [39,40]. Since the sandy grassland is a sensitive area for the occurrence and development of desertification in the semi-arid area, and the regional vegetation coverage and LST act as important indicators to identify the degree of local desertification [41]. Scale analysis of grid and sample area has important theoretical and practical significance for accurately understanding the influence of FVC on LST in this area.

### 4.1. Relationship between FVC and LST

From the comparison between the LST retrieved from Landsat8 images and the sLST, the LST extraction from Lansat8 in the study area is right in between the maximum sLST and minimum sLST of the corresponding period. From this point of view, the LST retrieved has a certain credibility (Table 1 and Figure 2 and Figure 3).

As indicated from the analysis of grid scale, the correlation analysis of FVC and LST in different groups (S1–S6, S7–S12, and S1–S12) showed the consistency of the results and achieved the purpose of mutual verification. To be specific, the correlation between FVC and LST was parabolic on 25 May, 10 June, and 29 August 2017. The spatial distribution maps of FVC and LST (Figure S1) in the Supplementary Materials can help to understand the above results. A positive correlation between FVC and LST was displayed when the FVC was less than 0.29–0.38, 0.27–0.32, 0.29–0.38 (turning point interval), respectively, as well as a negative correlation when FVC was larger than the above-mentioned interval. This is consistent with the results of Liu et al. [42], which showed that the cooling effect will be obvious only when the vegetation cover or green ecological land reached a certain proportion. Since sandy rangeland is composed of vegetation-covered and non-vegetation-covered areas (bare sandy land), the characteristics of LST would be determined by the warming effect dominated by bare land and the cooling effect dominated by vegetation. Before the turning point, bare sand patches distributed widely and became the dominant patches in the landscape, and the vegetation patches were small and scattered. When the vegetation coverage reached a certain proportion, the cooling effect of vegetation coverage would be stronger than the warming effect of sandy land. Such a phenomenon has also been verified in the study on urban landscape in different climatic zones [25,43]. In addition, a negative correlation was reported between LST and FVC on 28 July 2017, the warming effect of sandy land was at the maximum from the very beginning because the overall temperature of air and surface was at a high level (Table 1). Accordingly, the cooling effect of vegetation was dominant with the increase in FVC. This result was consistent with the conclusion of Karnieli et al. [44]. In the North American continent, when energy became the limiting factor of plant growth at the beginning of the growing season, there was a positive correlation between FVC and LST, while high temperature was the main factor leading to the negative correlation in the middle of the growing. In addition, vegetation responds to climate change, but shows a certain lag, which is one of the reasons for the diversification of the relationship between FVC and LST [24]. Indeed, there was another possibility that the specific heat capacity of sand was small, LST increased rapidly after absorbing heat, and the vegetation coverage hindered the surface heat dissipation, which would enhance the warming effect as well.

In addition, the ‘turning point’ of FVC-LST relationship showed to be slightly different at different temperature conditions (P1-P4). The higher the average air temperature and LST, the smaller the FVC when the inflection point occurs. For instance, the average air temperature (21.3 °C) and LST (40.8 °C) of 10 June 2017 was the highest in the study period except 28 July 2017, while the FVC was the smallest (0.26) when the turning point occurs. The cooling effects might also be related to the difference of other environmental factors like evapotranspiration at different stages of the growing [45].

**Figure 5:**
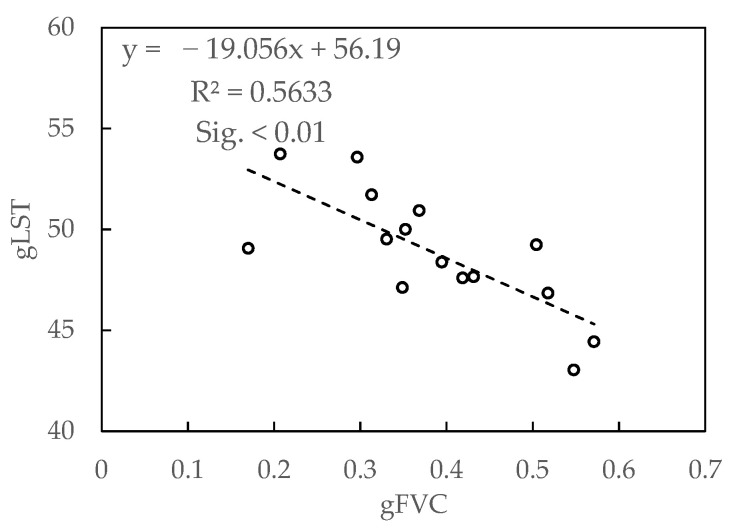
Relationship between gFVC and gLST at 23 July 2020 in field survey experiment.

At the sample area scale, the analysis of the basic characteristics of FVC showed that whether between P1 and P4 or 12 sample areas, FVC exhibited moderate or higher degree of variation, which demonstrated that the vegetation coverage gradient control and remote sensing data time selection in this study were of higher representativeness. At different temperature conditions, FVC and LST also showed positive and negative correlation, in which there was a positive correlation at P1, P2, P4, and a negative correlation at P3. This phenomenon was mainly determined by the level of FVC and air temperature [42]. It was indicated that the higher vegetation coverage showed an obvious cooling effect on the land surface at high air temperatures (Figure 3c), and when the air temperature was relatively low, the opposite was true (Figure 3a,b,d). This was consistent with the results on the grid scale, and mutual verification is realized. In addition, there was a negative correlation (*r* = −0.75) between gFVC and gLST (Figure 5) carried out by infrared thermometer (Raytek: RAYMX2C) in a circular sampling area of diameter 30 cm according to the field experiment at 10:40 am, 23 July 2020, in Naiman. The results were consistent with the scale of the sample area in July.

### 4.2. Spatial Pattern Correlation between FVC and LST

Given the classification of FVC and LST by the method of mean standard deviation, the landscape pattern metrics analysis revealed a good spatial matching (Figure 4, Table 6). Among the seven landscape indicators, SHEI, ENN_MN, and SPLIT indices showed a good correlation between FVC and LST, independent of temperature conditions. As indicated from the results, there was a spatial coupling between patch evenness, Euclidean nearest patch distance, and patch split degree. In addition, the other four landscape pattern metrics would be applicable at certain conditions. It will help to accurately determine the landscape distribution pattern under any condition and compare the differences under different conditions. At higher air temperature conditions and lower air temperature, the plaques of different grades of FVC and LST were aggregated, and AI showed a good linear fit with the above three landscape pattern metrics. However, at moderate air temperature, CONTAG index showed a good linear regression fitting with the above three landscape pattern metrics, demonstrating that different grades of FVC and LST patches were scattered at this condition. Therefore, the selection of landscape metrics and the results of the analysis will vary depending on the study area and the study period. Just as our study area was in a sandy area with a single surface cover type, the landscape distribution pattern of FVC and LST was highly matching.

On the whole, although the spatial distribution pattern of LST is more easily affected by the surrounding environment than vegetation, which shows complexity and uncertainty, the landscape pattern of FVC and LST still shows spatial coupling. Accordingly, from the application of FVC and LST in the identification of regional desertification degree, the near infrared band, red band and thermal infrared band of Landsat 8 could be exploited, and the selection of the appropriate landscape pattern metrics was also significant. Moreover, from the nature of the area we studied, there were almost two pure types, vegetation cover and no vegetation cover (bare sandy land), which avoided the use of urban areas or administrative districts as study units where LST may be influenced by other cover types around the vegetation [39].

Since vegetation and temperature are important indicators of desertification process, in which vegetation is the main factor affecting sand and dust movement, and the change of LST not only reflects the environmental condition, but also affects the growth of vegetation. By studying the diversity relationship between FVC and LST on different spatial scales, we can understand the interaction mechanism between them. Therefore, it not only plays an important role in monitoring the degree of desertification, but also provide theoretical reference for desertification control and vegetation ecological planning in semi-arid regions.

Finally, due the limit of time scale, spatial multi-scale analysis was only performed in our study and compared with the research result of Wu et al. [24]. To better understand the complex ecological correlations, multi-scale analysis methods must be adopted in future studies.

## 5. Conclusions

Based on the spatial multi-scale analysis of Landsat8 data, we proved the diversity relationship between FVC and LST. The results from May to August in Horqin Sandy Land showed that FVC had a significant cooling effect on LST when the FVC is more than 38% or the air temperature is more than 26.7 °C, and the opposite effect may occur under other conditions. The landscape evenness, Euclidean nearest neighbor distance, and landscape splitting degree of FVC and LST showed good correlation in any temperature condition, while some landscape patterns show coupling under specific condition. In the process of desertification, higher LST will aggravate land degradation, vegetation will not only achieve wind prevention and sand fixation, but also plays a role in cooling the surface. The analysis of this paper obtains the reference of the effective cooling interval brought by vegetation cover in sandy area, and the spatial coupling results of FVC and LST landscape pattern provides a theoretical basis for the planning and distribution of vegetation pattern at different temperature conditions.

## Figures and Tables

**Table 1:** Meteorological observatory data recorded by field meteorological stations corresponding to acquisition period of Landsat 8 images/(°C).

Station	Parameters	25 May 2017	10 June 2017	28 July 2017	29 August 2017
Naiman	air temperature	15.6	21.4	27.5	19.0
	maximum sLST	37.0	53.7	54.5	38.8
	minimum sLST	4.0	2.4	12.5	1.8
Kulun	air temperature	15.5	21.2	25.8	18.2
	maximum sLST	43.0	56.3	47.8	34.0
	minimum sLST	5.7	8.2	16.1	4.3

**Table 2:** Grading of FVC and LST at P1–P4.

	Grade	Interval	P1	P2	P3	P4
FVC	1st	fvc ≤ *μ_a_* ^1^ – *std_a_* ^2^	≤0.05	≤0.06	≤0.09	≤0.12
	2nd	*μ_a_* – *std_a_* < fvc ≤ *std_a_*	0.05–0.12	0.06–0.15	0.09–0.27	0.12–0.29
	3rd	*std_a_* < fvc ≤ *μ_a_* + *std_a_*	0.12–0.20	0.15–0.24	0.27–0.44	0.29–0.46
	4th	fvc > *μ_a_* + *std_a_*	>0.20	>0.24	>0.44	>0.46
LST/°C	1st	lst ≤ *μ_b_* ^3^ – *std_b_* ^4^	≤28.63	≤39.75	≤40.10	≤27.52
	2nd	*μ_b_* – *std_b_* < lst ≤ *std_b_*	28.63–30.27	39.75–40.8	40.1–41.68	27.52–28.62
	3rd	*std_b_* < lst ≤ *μ_b_* – *std_b_*	30.27–31.92	40.8–41.85	41.68–43.26	28.62–29.72
	4th	lst > *μ_b_ + std_b_*	>31.92	>41.85	>43.26	>29.72

^1, 2, 3^ and ^4^ are the average FVC, FVC standard deviation, average LST and LST standard deviation of the whole samples (S1–S12) in the study area on the condition of Pi (i = 1, 2, 3, 4), respectively.

**Table 3:** Landscape pattern metrics selected.

Landscape Pattern Metrics	Comments
NP (Number of patches)	The total number of patches of FVC (LST), expressing the landscape heterogeneity and fragmentation of FVC (LST).
LPI (Largest patch index)	The maximum patch area is divided by the total landscape area of the FVC (LST), expressing the landscape dominance.
ENN_MN (Euclidean nearest neighbor distance_Mean)	Nearest neighbor distance (average) between patches of FVC (LST).
SHEI (Shannon’s evenness index)	The landscape degree of uneven distribution of patches of FVC (LST).
SPLIT (Splitting index)	The landscape degree of segmentation and fragmentation of FVC (LST).
CONTAG (Contagion index)	The degree of aggregation or extension trend of patches with different grades of FVC (LST) in the landscape.
AI (Aggregation index)	Indicates the patches degree of dispersion and aggregation of FVC (LST).

**Table 4:** Representative sample areas selected at P1–P4.

Periods	Sample Area with Minimum FVC	Sample Area Closest to the Mean FVC	Sample Area with Maximum FVC
P1	S4	S2	S3
P2	S4	S10	S3
P3	S4	S5	S3
P4	S4	S8	S3

**Table 5:** Basic characteristics of FVC and LST in S1–S12 sample area at P1–P4.

Periods	FVC				LST/°C			
	Min_FVC	Max_FVC	Mean_FVC	*CV*/%	Min_LST	Max_ LST	Mean_LST	*CV*/%
P1	0.03	0.19	0.12	34.3	27.67	31.68	30.18	4.16
P2	0.04	0.25	0.15	37.0	39.80	42.07	40.80	1.62
P3	0.10	0.42	0.27	37.7	39.70	43.46	41.68	3.21
P4	0.12	0.45	0.29	33.5	27.11	29.44	28.62	2.66
*CV*/%	-	-	40.1	-	-	-	19.47	-

**Table 6:** Correlation coefficient (*r*) between FVC and LST landscape pattern metrics at P1–P4.

Sample Areas	P1	P2	P3	P4
Min_FVC	0.902 **	0.952 **	0.940 **	0.996 **
Median ^1^_FVC	0.725 *	0.989 **	0.892 **	0.748 **
Max_FVC	0.690 *	0.889 **	0.934 **	0.939 **

* indicates significant correlation at 0.05 level (double tail), ** indicates significant correlation at 0.01 level (double tail).^1^ are the representative sample areas closest to the mean FVC between S1–S12.

## Data Availability

Not applicable.

## Supplementary Materials

The following are available online at https://www.mdpi.com/article/10.3390/s21206914/s1. Figure S1: Spatial distribution maps of fractional vegetation coverage (FVC) and land surface temperature (LST) in S1–S12 sample area at four dates during the study period.

## Abbreviations

List of abbreviations (according to the order in which it appears)

Abbreviations	Definition
LST	Land Surface Temperature
NDVI	Normalized Difference Vegetation Index
FVC	Fractional Vegetation Coverage
UHI	Urban Heat Island
S1, S2, …, S12	Sample areas selected from study area in Figure 1
P1, P2, P3, P4	Period 1, 2, 3, 4, represents 25 May, 10 June, 28 July, 29 August, 2017, respectively.
NP	Number of Patches
LPI	Largest patch index
ENN_MN	Euclidean nearest neighbor distance_Mean
SHEI	Shannon’s evenness index
SPLIT	Splitting index
CONTAG	Contagion index
AI	Aggregation index
*CV*	Coefficient of variation
