# Supplementary material for: Spatial Scale Effects of the Relationship between Fractional Vegetation Coverage and Land Surface Temperature in Horqin Sandy Land, North China
Source: Sensors (Basel). 2021 Oct 19;21(20):6914. doi: 10.3390/s21206914 (PMC8539617; doi:10.3390/s21206914)
Supplement: Supplementary file 1 [file sensors-21-06914-s001.zip › sensors-1412542-supplementary.pdf]

**Figure S1.** Spatial distribution maps of fractional vegetation coverage (FVC) and land surface temperature (LST) in S1-S12 sample areas at four dates during the study period. May. represents 25th, May (P1); Jun. represents 10th, June (P2); Jul. represents 28th, July (P3); Aug. represents 29th, August (P4). The pixel values of the retrieved LST maps were standardized.

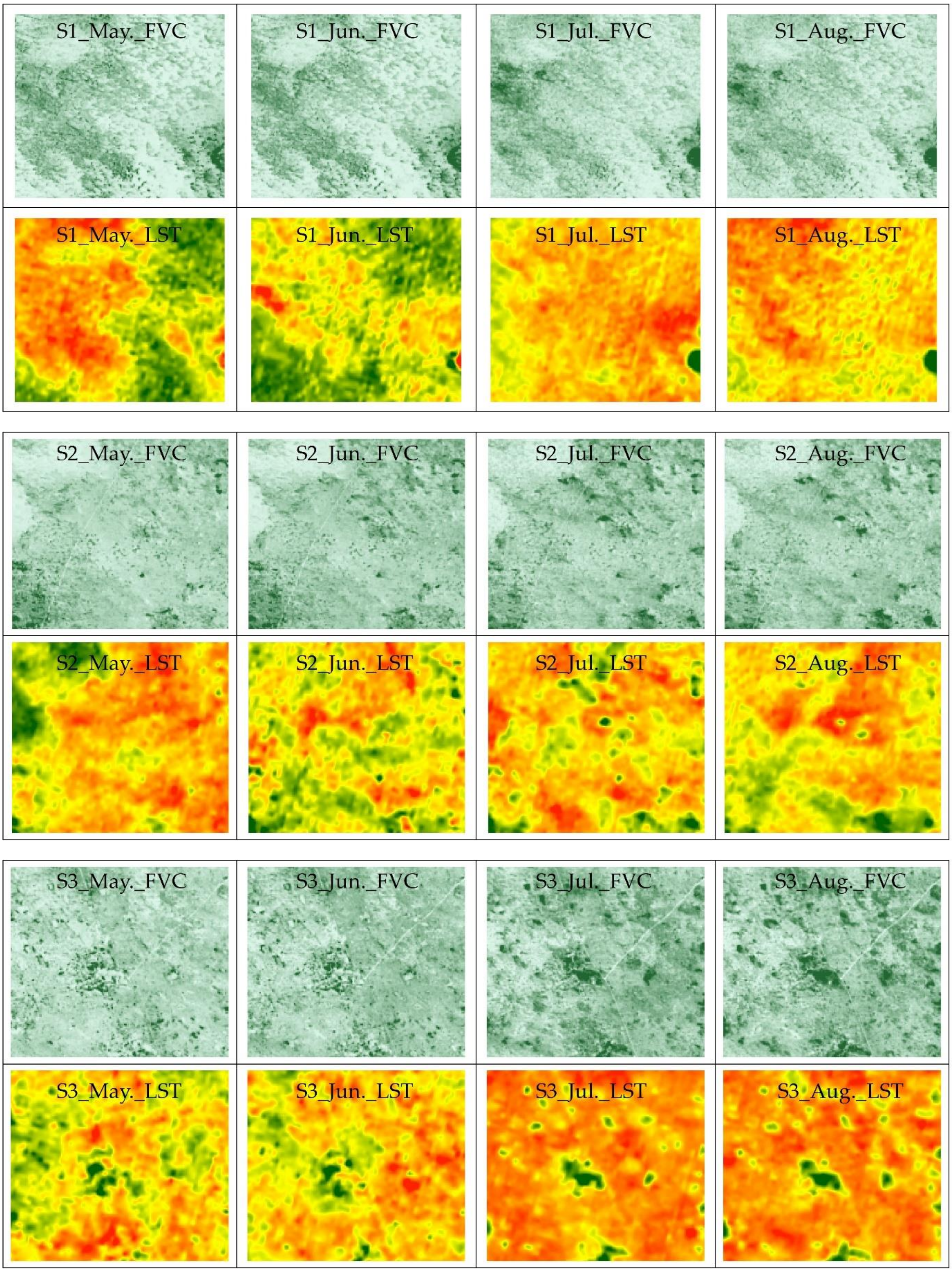

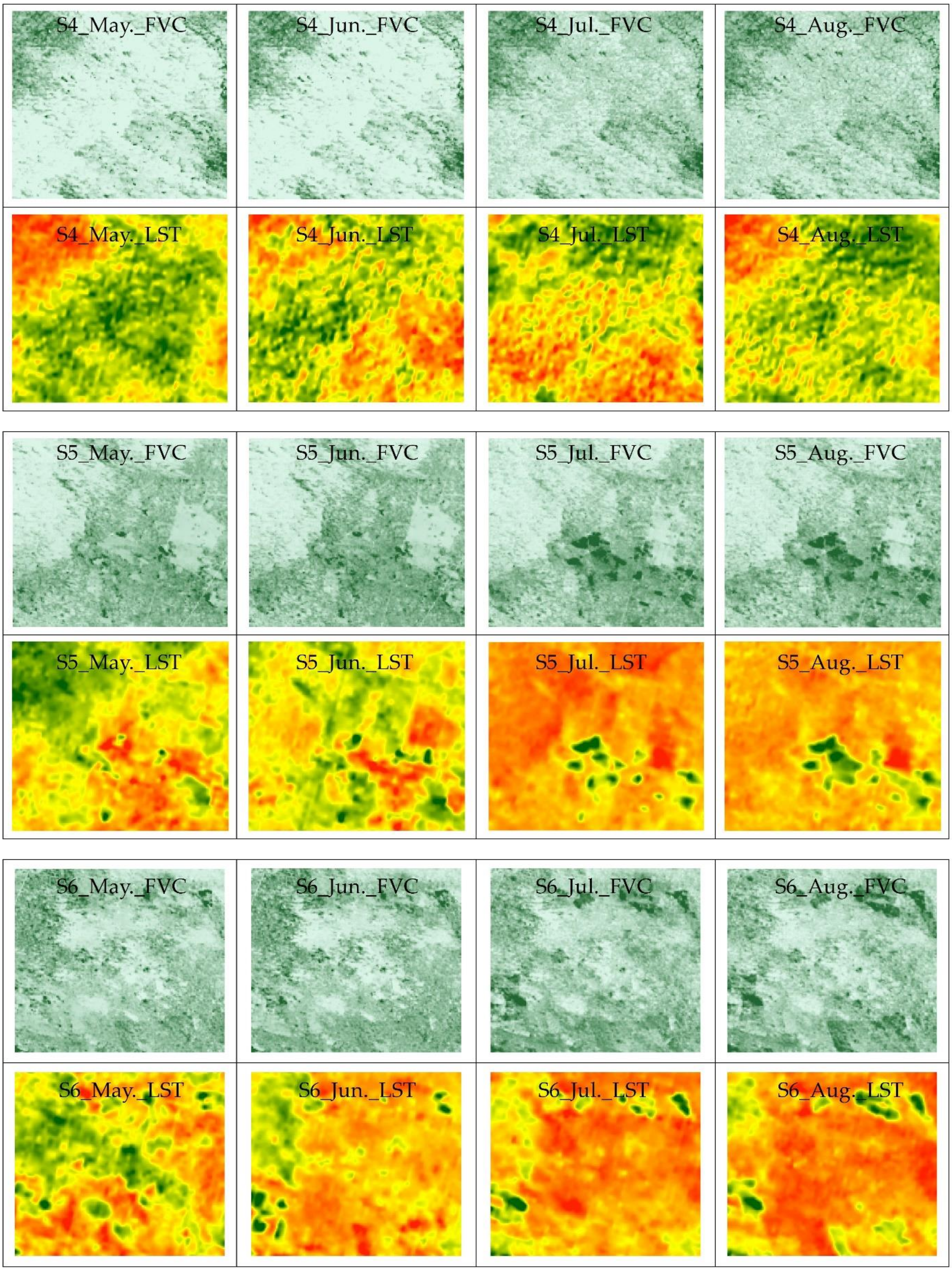
S4_May._FVC
S4_Jun._FVC
S4_Jul._FVC
S4_Aug._FVC
S4_May._LST
S4_Jun._LST
S4_Jul._LST
S4_Aug._LST
S5_May._FVC
S5_Jun._FVC
S5_Jul._FVC
S5_Aug._FVC
S5_May._LST
S5_Jun._LST
S5_Jul._LST
S5_Aug._LST
S6_May._FVC
S6_Jun._FVC
S6_Jul._FVC
S6_Aug._FVC
S6_May._LST
S6_Jun._LST
S6_Jul._LST
S6_Aug._LST

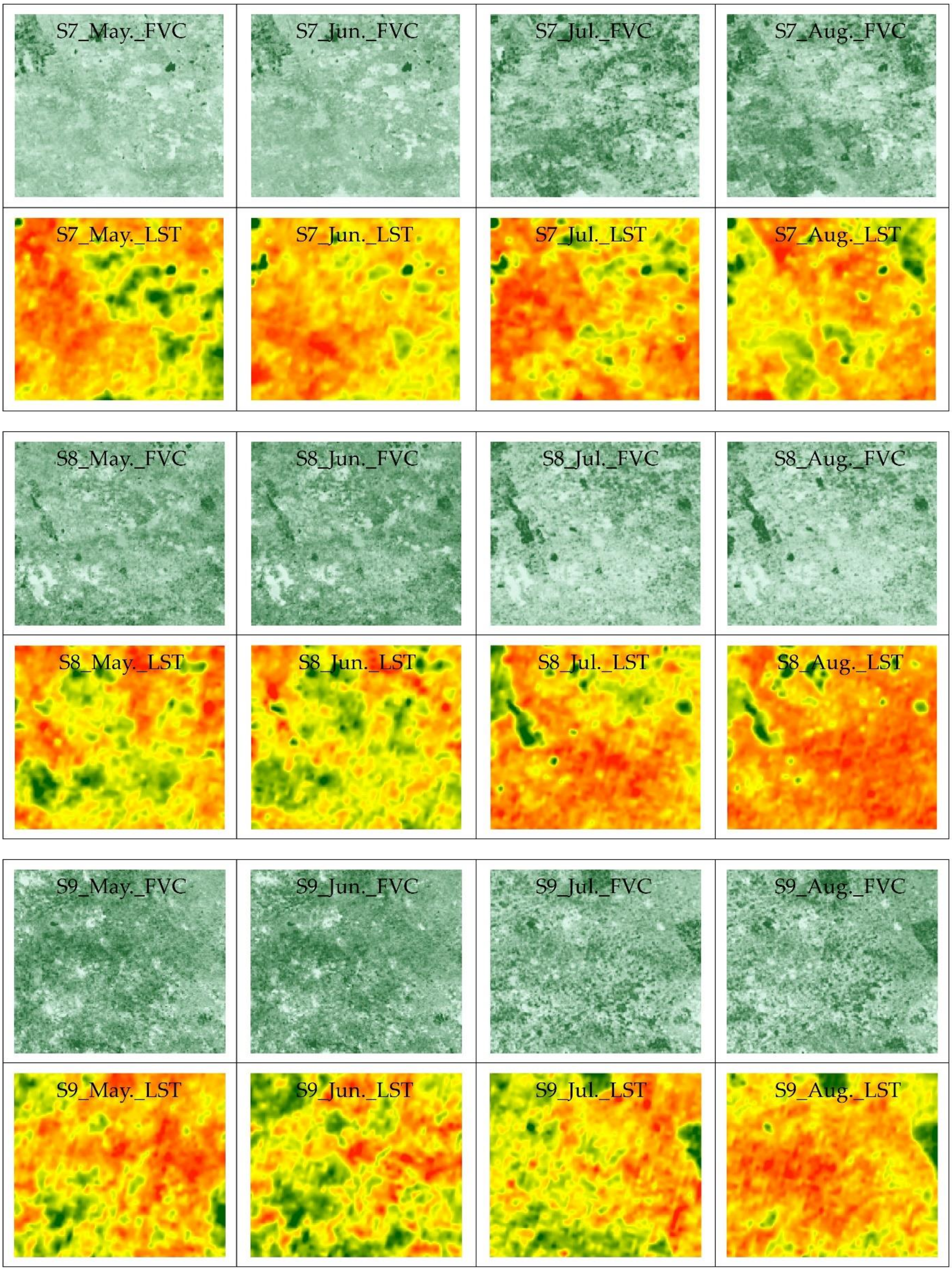
S7_May._FVC
S7_Jun._FVC
S7_Jul._FVC
S7_Aug._FVC
S7_May._LST
S7_Jun._LST
S7_Jul._LST
S7_Aug._LST
S8_May._FVC
S8_Jun._FVC
S8_Jul._FVC
S8_Aug._FVC
S8_May._LST
S8_Jun._LST
S8_Jul._LST
S8_Aug._LST
S9_May._FVC
S9_Jun._FVC
S9_Jul._FVC
S9_Aug._FVC
S9_May._LST
S9_Jun._LST
S9_Jul._LST
S9_Aug._LST

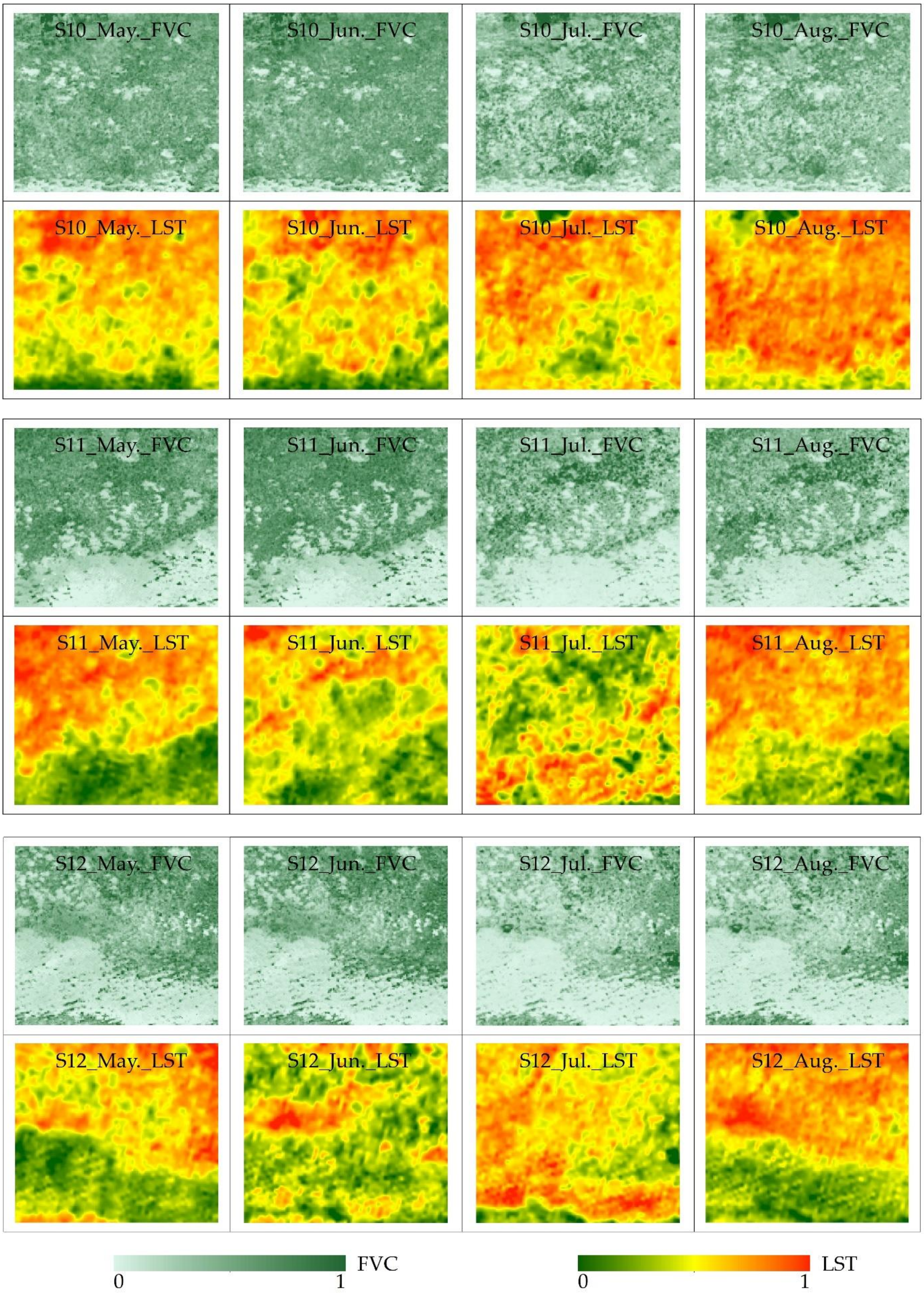
S10_May._FVC
S10_Jun._FVC
S10_Jul._FVC
S10_Aug._FVC
S10_May._LST
S10_Jun._LST
S10_Jul._LST
S10_Aug._LST
S11_May._FVC
S11_Jun._FVC
S11_Jul._FVC
S11_Aug._FVC
S11_May._LST
S11_Jun._LST
S11_Jul._LST
S11_Aug._LST
S12_May._FVC
S12_Jun._FVC
S12_Jul._FVC
S12_Aug._FVC
S12_May._LST
S12_Jun._LST
S12_Jul._LST
S12_Aug._LST
FVC
0
1
LST
0
1
